# Cancer care disparities among patients with limited English proficiency: challenges and strategies for equity

**DOI:** 10.1038/s44276-025-00187-6

**Published:** 2025-11-11

**Authors:** Hye Sung Kim, Carly Irwin, Arjun S. Ulag, Siddhartha Devarakonda

**Affiliations:** 1 Temple University Hospital, Philadelphia, PA USA; 2 Lewis Katz School of Medicine at Temple University, Philadelphia, PA USA; 3 Providence Swedish Cancer Institute, Seattle, WA USA; 4 Lakeside School, Seattle, WA USA; 5 Washington State University Elson S. Floyd College of Medicine, Spokane, WA USA

## Abstract

As the population of individuals with limited English proficiency (LEP) continues to rise in the United States, language barriers have become an increasingly important yet underrecognized driver of disparities in cancer care. This review aims to synthesize current evidence on how LEP affects the cancer care continuum and to offer actionable strategies to promote equity. We conducted a comprehensive review of the literature spanning communication, diagnosis, treatment, outcomes, prevention, research participation, and policy related to LEP populations in oncology. LEP is associated with poorer cancer outcomes, including delayed diagnosis, lower treatment adherence, decreased access to supportive services, and reduced quality of life. These disparities stem from multilevel communication barriers, underuse of professional interpretation, cultural discordance, and limited institutional support for language-concordant care. LEP patients are also underrepresented in cancer research due to language-based exclusion criteria, inadequate translation resources, and provider burden. A multifaceted framework is needed to address LEP-related disparities in oncology. Key strategies include expanding language-concordant care teams, improving interpreter and translation services, designing inclusive research protocols, and embedding language equity into institutional safety culture and policy. Addressing these disparities is a clinical, ethical, and public health imperative requiring systemic investment and leadership.

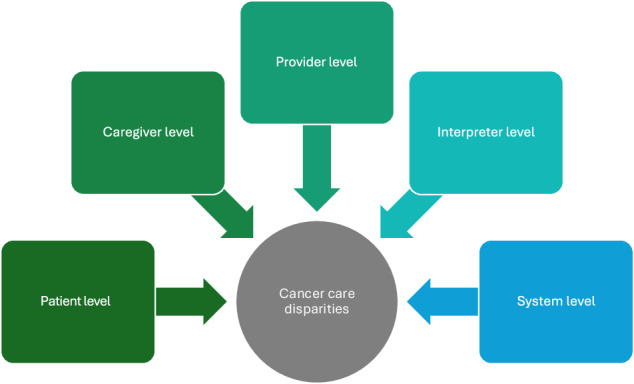

## Background

**Fig. 1:**
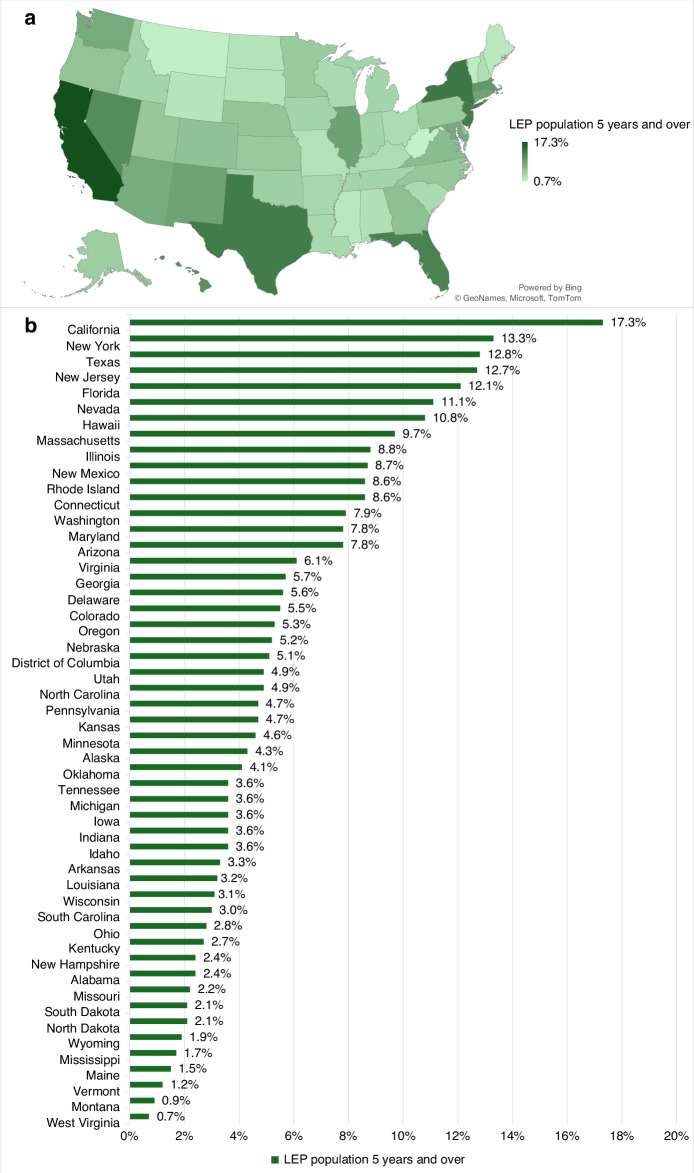
Geographic variation in limited English proficiency among U.S. population 5 years and over. **a** Choropleth map showing the percentage of U.S. residents aged 5 and older with LEP by state. Darker shades indicate states with a higher proportion of LEP individuals, ranging from 0.7% to 17.3%. **b** Bar graph ranking states by LEP prevalence among individuals aged 5 and older. California (17.3%), New York (13.3%), Texas (12.8%), and New Jersey (12.7%) report the highest proportions. LEP, limited English proficiency.

**Fig. 2:**
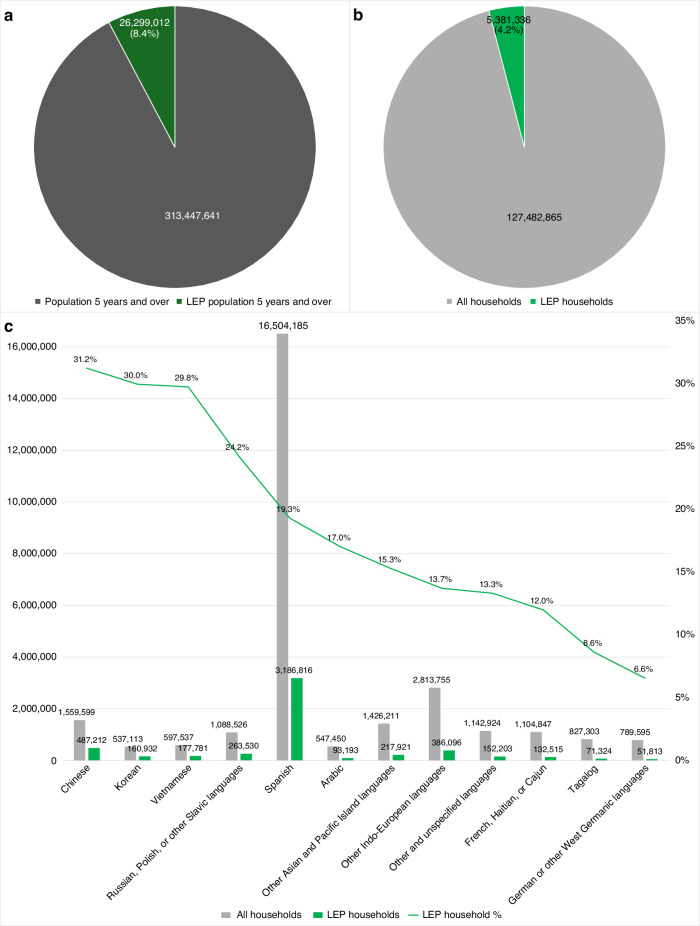
Population- and household-level variation in limited English proficiency in the U.S. **a** Proportion of U.S. residents aged 5 and older with LEP. Of 313.4 million individuals, 26.3 million (8.4%) are classified as LEP. Abbreviations: LEP, limited English proficiency. **b** Proportion of U.S. households in which no member aged 14 or older (1) speaks only English or (2) speaks a non-English language and speaks English “very well.” Of 127.5 million households, 5.4 million (4.2%) meet the LEP household criteria. **c** Distribution of LEP households by primary language spoken. Gray bars represent the total number of U.S. households per language group, green bars represent LEP households within each group, and the green line indicates the percentage of LEP households within that group. Spanish-speaking households account for the largest share (3.2 million), while Chinese (31.2%), Korean (30.0%), and Vietnamese (29.8%) households exhibit the highest within-group LEP rates. Abbreviation: LEP, limited English proficiency.

As of 2023, the U.S. immigrant population reached a record high of 47.8 million [1]. Among individuals aged 5 and older, 68.8 million (22.0%) speak a language other than English at home, with Spanish being the most common (13.4%) [2]. An estimated 26.3 million individuals (8.4%) and 5.4 million households (4.2%) are considered limited English proficient (LEP), indicating difficulty with reading, writing, speaking, or understanding English (Figs. 1 and 2).

The growing LEP population raises critical concerns about language-related disparities in healthcare. Language barriers impede effective communication with medical staff, hinder understanding of care instructions, and create difficulties with form completion, medication management, and appointment scheduling [3]. Compounding these challenges, many LEP individuals face lower income, limited educational attainment, and employment in low-wage jobs, factors that increase the likelihood of being uninsured and exacerbate disparities in access, adherence, and preventive care [4–7]. Together, these systemic barriers contribute to poorer health outcomes, including inadequate chronic disease management, greater reliance on emergency department (ED) services, longer hospital stays, and higher rates of readmissions and complications [4–7].

Despite growing research on healthcare disparities, the impact of LEP on cancer care remains underexplored. Most data come from English-speaking populations, with limited attention to language barriers, inconsistent reporting, and few targeted interventions [8, 9]. As the burden of cancer rises, especially among racial and ethnic minorities, language discordance remains a persistent barrier to effective care, even with federal protections in place [10]. This review outlines the unique challenges LEP patients face in cancer care, highlights disparities across the continuum, and offers actionable strategies to promote language-concordant care and improve outcomes for this underserved population.

## Communication disparities across the cancer care continuum

Challenges in communication among patients, caregivers, providers, interpreters, and at the system level contribute significantly to disparities in cancer care for LEP individuals. Patients with LEP often lack awareness of available support, such as free interpreter services, and rely on family members for translation, raising concerns about privacy and accuracy [11]. Access to language-specific materials (e.g., discharge instructions or treatment plans) is limited, and when available, they are frequently overly technical, culturally insensitive, or lacking actionable guidance (e.g., clinic contact information or referrals) [12–14].

These challenges are compounded by the complexity of the U.S. healthcare system. LEP patients may be unfamiliar with shared decision-making norms and may hold cultural beliefs (such as *respeto* and *fatalismo* among Hispanic populations) that discourage questioning providers [15–17]. When combined with low health literacy and limited information-seeking behaviors, these factors can lead to reduced engagement in care and poor adherence to treatment recommendations [11]. LEP patients also face systemic barriers to financial support and may be less likely to receive assistance, further widening existing disparities [18, 19]. Caregivers encounter similar barriers, which may be intensified by undocumented immigration status, financial strain, and limited access to psychosocial resources [18, 20, 21].

On the provider side, language barriers lead to incomplete histories, inadequate consent processes, and missed opportunities for patient education [22]. Reliance on untrained *ad hoc* interpreters and underuse of professional services, due to time pressure or overestimation of language proficiency, further compromise care [11, 13, 23, 24] Even professional interpreters may lack the training for complex topics like clinical trials or genetic counseling, leading to errors and miscommunication [13, 25–28]. At the system level, limited interpreter availability, cost barriers, and interpreter delays impede timely, high-quality care [11, 29]. Language barriers are particularly harmful during critical conversations, including end-of-life discussions, where they are linked to poorer quality of life outcomes [30].

National data highlight substantial gaps in institutional compliance with language access standards. In a survey of Medicare providers, 69% reported conducting the four-factor assessment recommended by the Office for Civil Rights, yet only 33% offered services consistent with all four National Culturally and Linguistically Appropriate Services (CLAS) Standards [31]. While 73% acknowledged benefits to providing language access services, nearly half cited obstacles, such as staffing shortages and equipment limitations, and few could provide reliable data on costs [31]. These findings underscore that interpreter and translation services remain inconsistently implemented, with persistent technical barriers and resource constraints reinforcing disparities and hindering equitable cancer care for LEP patients.

## Disparities in cancer detection and care delivery

Language discordance contributes to disparities in both cancer incidence and stage at diagnosis. States with high LEP populations, such as California (8.5%) and New York (7.6%), report elevated rates of ovarian and stomach cancers: 10.4 and 9.1 per 100,000 in California, and 11.3 and 10.9 in New York, respectively [32]. LEP patients are also more likely to present with advanced-stage cancers, including pancreatic, head and neck, and breast cancers, as well as higher-risk pediatric malignancies [13, 33–36]. While some studies note lower odds of late-stage colorectal cancer among Hispanics, the broader evidence highlights diagnostic delays tied to language barriers [37].

These diagnostic disparities extend into treatment and follow-up. LEP patients, especially Spanish-speaking and uninsured individuals, experience delays in initiating care for breast, head and neck, colorectal, and lung cancers [38–40]. These delays typically stem from difficulty understanding pre-treatment instructions, managing medications, monitoring symptoms, and navigating follow-up. Although one study found no language effect on radiotherapy timing, LEP remains associated with reduced receipt of cancer-directed therapies and variable treatment plans, particularly in pancreatic and head and neck cancers [33, 34, 41, 42].

During follow-up, LEP patients, especially in pediatrics, show lower adherence to survivorship care and more frequent missed visits [13, 43]. They also have higher ED use, longer hospital stays, and greater readmission rates, particularly in breast, pancreatic, and brain cancers [9, 13, 44–47]. Higher weekend admission rates suggest delayed care-seeking and more advanced illness [48, 49]. LEP is also independently associated with increased healthcare costs and higher likelihood of discharge to skilled nursing facilities [50].

## Disparities in clinical outcomes and quality of life

Despite treatment, patients with LEP may experience worse clinical outcomes. Those with head and neck cancer, for example, have poorer locoregional control following radiation, likely due to missed appointments, misunderstanding of protocols, or underreporting of side effects [51]. Difficulty understanding medication labels and pre-surgical instructions can also lead to dosing errors, improper fasting, and missed warning signs, increasing the risk of serious complications [5, 52, 53].

LEP patients frequently report lower quality of life, higher symptom burden, and greater emotional distress during cancer care. These issues are often rooted in miscommunication surrounding diagnoses, treatment plans, discharge instructions, and follow-up care [54–56]. While some studies show no significant differences in hospice or palliative care use, language barriers continue to hinder effective end-of-life communication and decision-making [50, 57–59]. In pediatric hospice, Spanish-speaking families have described frustrations with insensitive communication and distressing ED interactions [60]. Insurance challenges further restrict access to supportive services, such as hospice care, visiting nurses, and specialty referrals [11].

Survival disparities have also been documented among LEP patients, particularly in breast, pancreatic and pediatric cancers, where lower overall, disease-free, and disease-specific survival have been observed [13, 33, 38]. However, findings are mixed; some studies show no differences, and certain groups, like Haitian Creole speakers with prostate cancer, have even shown improved survival [45, 61–63]. These exceptions may be suggestive of the underlying differences in institutional resources and healthcare infrastructure and present opportunities for targeted interventions [48, 64].

## Disparities in prevention and early detection

Language barriers, limited familiarity with preventive care, and systemic inefficiencies reduce awareness and participation in services, such as immunization, genetic counseling, and cancer screenings [65]. English proficiency strongly correlates with HPV awareness, and misconceptions about vaccine-preventable cancers remain prevalent among those with LEP [66]. While LEP patients may access genetic counseling for breast cancer at similar rates to English speakers, reliance on family members as intermediaries can lead to filtered information and loss of patient autonomy [67, 68]. In some cases, family members even withhold diagnoses, complicating decision-making.

LEP is consistently linked to lower cancer screening rates. Urban LEP populations, such as in New York City, show reduced colorectal and cervical screening [69]. Asian Americans with LEP are less likely to be up-to-date on breast, cervical, and colorectal screenings [70–72]. Clinics unequipped for translation services exacerbate delays in cervical screening [73]. LEP patients also demonstrate lower completion rates for colorectal, prostate, and lung cancer screenings [74–77].

## Disparities in cancer research participation

LEP individuals remain underrepresented in clinical trials, with enrollment stagnating despite a growing population, particularly in gynecologic oncology [67, 78, 79]. Many U.S. interventional trials exclude non-English speakers, with 40% citing reasons such as unanticipated language needs (51%), difficulty translating materials, and lack of bilingual staff [13, 80, 81]. Limited translated resources and financial constraints further restrict access, disproportionately burdening LEP patients [82]. Notably, when funding for translated consent forms was withdrawn in gynecologic oncology trials, enrollment of Hispanic LEP patients sharply declined [83].

Provider-related factors compound these challenges. Many clinicians may find it burdensome to determine eligibility (75%) or explain trial risks and benefits (68%) to LEP patients [84]. As a result, essential details, such as randomization, withdrawal rights, and consent documentation, are often poorly communicated, leaving patients less informed and deepening mistrust [16, 84–86]. This underrepresentation skews data on drug efficacy and safety, limiting the generalizability of trial findings and reinforcing disparities.

## Strategies to address disparities in cancer care

**Fig. 3:**
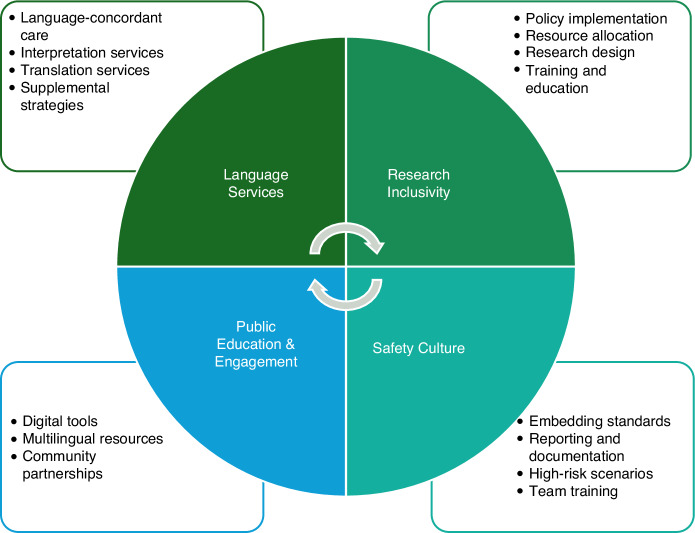
Key domains for addressing cancer care disparities in individuals with limited English proficiency. Framework highlighting four core domains for reducing cancer care disparities among individuals with LEP: (1) Language Services, including language-concordant care, interpretation, translation, and supplemental strategies; (2) Research Inclusivity, focusing on equitable policy implementation, resource allocation, inclusive study design, and training; (3) Public Education & Engagement, involving digital tools, multilingual resources, and community partnerships; and (4) Safety Culture, emphasizing standardization, reporting, high-risk scenario planning, and team-based training. Abbreviation: LEP, limited English proficiency.

Addressing cancer care disparities among patients with LEP requires a multifaceted strategy (Fig. 3). Here, we discuss and propose interventions to improve access, quality, and outcomes across the cancer care continuum.

### Language access policies

**Fig. 4:**
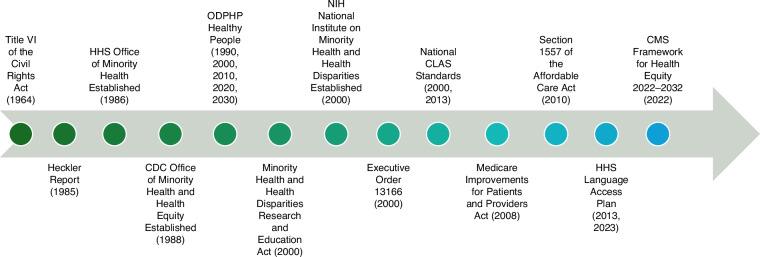
Timeline of major U.S. policies and initiatives advancing health equity and language access. Timeline highlighting key federal policies, reports, and initiatives from 1964 to 2023 that have shaped efforts to reduce health disparities and improve language access for individuals with LEP. Abbreviations: CDC, Centers for Disease Control and Prevention; CLAS, Culturally and Linguistically Appropriate Services; CMS, Centers for Medicare & Medicaid Services; HHS, U.S. Department of Health and Human Services; LEP, limited English proficiency; NIH, National Institutes of Health; ODPHP, Office of Disease Prevention and Health Promotion.

Language access is a civil right rooted in principles of distributive and language justice, ensuring equitable resource allocation and affirming the dignity of linguistic minorities. Creating multilingual spaces enables LEP individuals to fully participate in care and benefit from medical advancements. Over time, key policies have laid a foundation for addressing disparities in cancer care (Fig. 4). By 2019, all U.S. states and D.C. had enacted healthcare language access laws. California leads with 257 provisions, including Medicaid translation requirements [87]. While all states have at least three provisions, their scope varies, ranging from broad mandates to targeted measures for specific providers or populations. Some include detailed implementation guidance; others recognize language access without specifying actions.

Together, these policies form an ethical, legal, and practical framework to reduce disparities. Recognizing language access as a civil right ensures LEP patients are not excluded from health equity efforts. Embedding equity principles in cancer care requires a coordinated, ongoing effort from policymakers and healthcare institutions alike.

### Language services

Recognizing language access as a civil right is foundational, but effective implementation requires practical strategies. One such strategy for expanding language-concordant care is to involve providers, navigators, and community health workers who can speak a patient’s preferred language, when feasible.

### (1) Language-concordant teams

Language-concordant providers improve satisfaction, communication, and therapeutic relationships [88, 89]. Among Asian Americans, those with language-concordant clinicians reported screening rates comparable to English speakers, highlighting its impact on preventive care [70]. Conversely, the shortage of Spanish-speaking providers contributes to disparities for Hispanic/Latino patients [90]. Culturally competent nurses help patients navigate the U.S. healthcare system, encouraging autonomy and symptom reporting [11]. Patient navigators have successfully recruited and retained underserved Latino adults with advanced cancer in a randomized trial and increased cancer screening rates across multiple populations [91–94]. Community health workers, such as *promotoras* in Hispanic communities, further boost screening rates and health literacy through culturally tailored, language-aligned interventions [71, 95–100].

In more specialized contexts, language-concordant genetic counselors have been instrumental in improving patient experiences during cancer genetic counseling. Spanish-speaking patients report positive outcomes when counseled in their preferred language, emphasizing the need for linguistic alignment in complex discussions about hereditary cancer risks [101]. Likewise, language-concordant patient family advocates in pediatric oncology have reduced stress and fostered trust, communication, and continuity of care for LEP families [101, 102].

To expand the availability of language-concordant care, several workforce strategies have been proposed. These include targeted recruitment and retention of bilingual medical students and residents, supported by loan forgiveness or pipeline programs aimed at underrepresented linguistic groups, and incentivizing dual certification, such as formal Spanish proficiency testing with CME credit [103]. Embedding community health workers and *promotoras* directly into oncology care teams ensures culturally aligned navigation throughout the cancer care continuum, while leveraging telemedicine can connect LEP patients to language-concordant specialists across institutions, particularly in settings where local bilingual providers are unavailable [104, 105].

### (2) Interpretation services

Full language concordance is not always feasible due to resource constraints, workforce shortages, and linguistic diversity. In such cases, interpretation services play a critical role in bridging communication gaps. Trained interpreters improve comprehension, reduce errors, and enhance clinical outcomes and satisfaction compared to *ad hoc* alternatives [106–108]. In pediatric oncology, interpreters frequently extend beyond their formal roles to support families during complex care [109].

Remote options via telephone and video expand access and timeliness. Studies show they can match or exceed in-person interpreting for routine tasks like discharge instructions [110–114]. Bilingual phone interventions have increased cancer screening, especially among Spanish-speaking women, and improved Hispanic enrollment in clinical trials [115, 116]. Despite their benefits, providers often prefer in-person interpreters for complex discussions (e.g., device education, advanced care planning) due to the lack of visual cues, background noise, connectivity issues, and limited dialect support with remote options [24, 117].

To optimize interpretation services, hospital administrations should prioritize funding for interpreter infrastructure and training [118–120]. They are well-positioned to expand remote options, implement automated scheduling systems that streamline access for LEP patients, and ensure the delivery of standardized interpreter training programs. Requiring at least 100 h of training for interpreters has been shown to reduce clinical errors by up to 70% and significantly improve safety and quality [27, 107, 121]. These programs should also incorporate pre- and post-encounter briefings to align expectations and address challenges [122, 123]. Ultimately, apart from hospital administrations, the responsibility for proper implementation of interpreter services must be shared across different stakeholders: nursing leadership could oversee the implementation of training modules, while professional oncology societies and accrediting bodies can embed language-access standards into quality benchmarks. Frontline clinicians and nurses must also be educated to consistently document interpreter use in the electronic health record [124, 125].

### (3) Translation services

While interpretation supports real-time communication, translation services are vital for providing LEP patients with accessible written materials throughout cancer care. One key application is improving medication adherence. The University of Colorado Health translated prescription labels into 26 languages, enhancing understanding and adherence among non-English speakers [126]. Similarly, ConcordantRx used health literacy-informed multilingual instructions to improve dosing accuracy and treatment adherence [127]. Yet, many pharmacies still rely on patient requests. Proactively offering multilingual labels and counseling patients on medication use can significantly enhance safety [128].

Digital tools further expand access. Apps like MediBabble and platforms like Google Translate help providers and LEP patients communicate in urgent settings, improving satisfaction [5, 129–131]. Tools like Sisom help children express cancer symptoms in their native language [132]. However, these tools often lack accuracy for complex medical content and should supplement, not replace, professional translation [129, 130]. Federal guidelines stress that machine-generated content must be reviewed by qualified human translators to meet legal standards [133–135].

To optimize translation services, systems must ensure accuracy, address cost barriers, and proactively offer multilingual resources. Providers play a key role in assessing comprehension and tailoring materials. Moffitt Cancer Center demonstrates this with an integrated model offering free interpreting and translation services. Between 2011 and 2013, they completed over 25,000 interpretation encounters and 1400 translation projects, earning a 4.8/5 patient satisfaction score [136].

### Inclusive research

Sponsors and pharmaceutical companies have a critical role in addressing the underrepresentation of LEP individuals in clinical research. Recommended strategies include mandating dedicated budget lines for translating consent forms and other trial materials, requiring sites to demonstrate interpreter access before trial activation, partnering with community-based organizations for multilingual outreach, and publicly reporting enrollment demographics stratified by language to enhance transparency [137, 138]. Some sponsors have piloted initiatives, such as multilingual enrollment hotlines and the hiring of bilingual research coordinators, which have shown promise in improving participation among LEP patients [138]. Yet these remain isolated examples; broad implementation of such measures is critical to achieving equity goals in clinical trials.

Federal agencies emphasize the ethical importance of inclusive research. The Food and Drug Administration (FDA) recommends that Institutional Review Boards (IRBs) ensure translated consent materials and interpreter support throughout the trial [139]. Informed consent should be treated as an ongoing dialogue, not a one-time event. However, IRB practices vary; some offer comprehensive language access guidelines, while others do not [140]. Initiatives such as the FDA’s Section 907 Action Plan and Drug Trials Snapshots aim to improve demographic representation and highlight differences in treatment outcomes across subgroups [141, 142].

To further promote inclusivity, IRBs should avoid imposing language restrictions unless justified [80]. Journals can support this goal by requiring researchers to explain their inclusion and exclusion criteria [143]. Equipping investigators with the skills to collaborate with interpreters and design linguistically inclusive protocols is critical, and clinicians play a key role in advocating for language access in both clinical care and research settings [137, 138]. Ultimately, creating a more inclusive research environment is not only an ethical and legal responsibility; it is a scientific necessity for producing valid, generalizable findings that reflect the populations served.

### Safety culture

Creating a culture of safety for LEP patients requires healthcare organizations to embed language access and cultural competence into every level of care. Language barriers significantly increase the risk of adverse events, underscoring the need to incorporate these priorities into safety protocols, supported by leadership, strategic planning, and dedicated resources. The Agency for Healthcare Research and Quality emphasizes that overcoming language barriers should be central to an institution’s quality and safety mission [144]. Embedding language access into operational policies and learning from LEP-related safety events promotes continuous improvement. Patient and family advisory councils offer important perspectives for guiding targeted interventions.

A strong safety culture also relies on accurate reporting and documentation. Staff, including interpreters, should be trained to report all safety events, including near misses. The FDA’s MedWatch program supports this process with Spanish-language reporting tools [145]. Documenting language preferences and interpreter use in electronic health records enables health systems to monitor outcomes and identify areas for improvement. Certain clinical scenarios, such as medication reconciliation, discharge, informed consent, emergency care, and surgery, pose heightened risks for LEP patients. These moments require qualified interpreters and plain-language translated materials to ensure clarity and prevent miscommunication. The teach-back method can be used to confirm patient understanding.

To support ongoing improvement, organizations should implement structured team training. The Agency for Healthcare Research and Quality’s TeamSTEPPS LEP Module equips interdisciplinary teams to enhance communication and reduce errors [146]. Techniques like briefs, check-backs, and teach-backs foster collaboration and psychological safety among care teams. Ultimately, fostering a robust safety culture for LEP patients requires institutional commitment, regular review of LEP-related data, and sustained investment in language-access infrastructure.

### Public education and engagement

Public education is essential for meeting the needs of LEP patients by improving health literacy, navigation, and access to services. Multilingual resources help LEP individuals navigate healthcare systems and address social determinants of health. LEP.gov (available in 21 languages) and the Coverage to Care initiative (in nine languages) provide guidance on coverage, primary care, and preventative services.

Public education also improves cancer literacy. Tools like The Health Communicator support older migrant patients with tailored, multilingual resources. NCCN Guidelines®, available in multiple languages and mobile formats, empower patients and clinicians alike. Language-concordant platforms, such as call centers and websites, boost engagement in clinical trials [147]. Digital tools assist LEP patients in tracking symptoms and managing care. The American Cancer Society’s Personal Health Manager (English/Spanish) and Spanish-language electronic symptom reporting systems improve communication and care continuity [148, 149]. Multimedia interventions, including native-language videos, reminders, and culturally adapted materials, have increased screening rates among refugees and LEP populations [100, 150–152]. A Florida cancer center’s multilevel strategy combining interpreters, navigators, and provider training notably boosted lung cancer screening among Hispanic and Black patients [153].

Partnerships with community organizations enhance outreach. FDA-led webinars and advisory committees give LEP communities a voice in health policy. Biweekly updates from the FDA’s Office of Minority Health and Health Equity keep patients and providers informed about safety, funding, and research initiatives.

## Conclusion

Disparities in cancer care for LEP patients span the entire care continuum from prevention to survivorship. This review highlights the need for comprehensive, multifaceted strategies to close these gaps. Key approaches include expanding language-concordant care, improving interpretation and translation services, and enhancing public education and engagement. Tailored digital tools, multilingual materials, and culturally adapted interventions have shown success in improving cancer literacy, screening uptake, and treatment adherence. However, these efforts must be paired with systemic changes, such as equitable digital access, targeted workforce training, and strong policy enforcement to ensure sustained impact. Partnering with community organizations, stakeholders, and LEP individuals creates a feedback loop that builds trust, improves communication, and informs culturally relevant programs and policies.

Ultimately, addressing these disparities is both a moral and public health imperative. By advancing inclusive, patient-centered care and embedding equity and safety into healthcare systems, we can ensure all patients, regardless of language proficiency, receive high-quality, compassionate cancer care.

## Data Availability

The data supporting Figs. 1 and 2 are publicly available from the U.S. Census Bureau, 2019-2023 American Community Survey (ACS) 5-Year Estimates. Data can be accessed through the Census Bureau’s data portal at: https://data.census.gov/. No additional proprietary datasets were generated or analyzed for this study.
